# A novel fibrinogen mutation: *FGA* g. 3057 C > T (p. Arg104 > Cys) impairs fibrinogen secretion

**DOI:** 10.1186/s12878-017-0086-8

**Published:** 2017-12-22

**Authors:** R. Marchi, M. Linares, H. Rojas, A. Ruiz-Sáez, M. Meyer, A. Casini, S.O. Brennan

**Affiliations:** 1 Lab. Biología del Desarrollo de la Hemostasia. Instituto Venezolano de Investigaciones Científicas (IVIC), Caracas, Bolivarian Republic of Venezuela; 2 Instituto de Inmunología, Universidad Central de Venezuela and Lab. Fisiología Celular Centro de Biofisica y Bioquímica (IVIC), Caracas, Bolivarian Republic of Venezuela; 3 Banco Municipal de Sangre del Distrito Capital, Caracas, Bolivarian Republic of Venezuela; 4 Medical Engineering and Biotechnology, University of Applied Sciences, Jena, Germany; 5 Division of Angiology and Haemostasis, Faculty of Medicine, University Hospitals of Geneva, Geneva, Switzerland; 6 Molecular Pathology Laboratory, University of Otago, Christchurch, New Zealand

**Keywords:** Abnormal fibrinogen, Hypodysfibrinogenemia, Fibrinogen Aα chain

## Abstract

**Background:**

Abnormal fibrinogens can be caused by clinically silent hereditary mutations. A new case was detected accidentally in an 11-year-old girl when routine pre-operative coagulation tests were performed for nasal turbinate surgery.

**Methods:**

The fibrinogen genes FGA, FGG and FGB were sequenced using standard protocols. The kinetics of fibrin formation were followed by turbidity at 350 nm. Purified fibrinogen was incubated with plasmin, and the degradation products analyzed by SDS/PAGE. The formation of fibrinogen-albumin complexes was analyzed by immunobloting. Fibrin structure was examined in a Nikon Eclipse TE 2000-U laser microscope. Secretion of the variant protein was analyzed directly by reverse phase-electrospray time of flight-mass spectrometry (TOF-MS).

**Results:**

DNA sequencing revealed a novel heterozygous g. 3057 C > T mutation in the *FGA* that predicts a p. Arg104 > Cys substitution, in the proband and her father. Both patients were asymptomatic with low functional and antigen fibrinogen concentrations. The proband’s plasma fibrinogen polymerization was almost normal, with a 12% decrease in the final turbidity, while, the father’s fibrin formation had a diminished slope and final turbidity (2.5× and 40%, respectively). Aα Arg104 is located at a plasmin cleavage site in the coiled-coil region of fibrinogen. However, the father’s fibrinogen plasmin degradation was normal. Although the exchanged Cys introduces an unpaired –SH, immunoblotting showed no fibrinogen-albumin complexes. Furthermore, the plasma clot structure observed by confocal microscopy appeared almost normal. TOF-MS showed that the variant Aα chain was underrepresented in plasma and made up only about 25% of the total.

**Conclusions:**

The low expression of the Aα Arg104 > Cys chain in circulation could account for the observed hypodysfibrinogenemia.

**Electronic supplementary material:**

The online version of this article (10.1186/s12878-017-0086-8) contains supplementary material, which is available to authorized users.

## Background

Fibrinogen is the central protein of blood coagulation. Once the coagulation cascade is initiated, thrombin is formed and catalyzes the conversion of fibrinogen into soluble fibrin monomers that polymerize spontaneously, forming a three-dimensional network that becomes further stabilized by activated factor XIII (FXIIIa). Polymerization is initiated by cleavage of the A and B peptides from the N-terminal of the Aα and Bβ chains [1]. Fibrinogen is a 340 kDa glycoprotein synthetized in the liver and normally circulates in plasma at 160–400 mg/dl [2]. It is composed of two sets of three different polypeptides chains (Aα, Bβ,γ)_2_, arranged in three nodules: the N-terminal of the six chains converge at the center forming the globular E region. A coiled coil of all the three chains extends from each side of the E domain to connect with the outer D domains, which form from the C-terminal region of the Bβ and γ chains. The coiled coil is delineated by two disulfide rings and its central region has a kink in its structure that acts as the primary attack site for plasmin [3].

Inherited fibrinogen disorders can be quantitative (Type I; absence or decreased level of circulating fibrinogen, afibrinogenemia and hypofibrinogenemia, respectively) or qualitative (Type 2; normal or decreased antigenic levels and low fibrinogen activity, dysfibrinogenemia and hypodysfibrinogenemia, respectively) [2, 4].

Dysfibrinogenemia is caused by structural abnormalities that can be inherited (congenital) or acquired [5]. Inherited dysfibrinogenemia is caused by mutations in the coding region of the fibrinogen Aα, Bβ or γ genes and the majority of cases result from heterozygous missense mutations [4]. The prevalence of inherited dysfibrinogenemia in the general population is unknown [5]. The pattern of inheritance is autosomal dominant, and 55% of the patients are asymptomatic, while 25% develop bleeding or thrombosis. Hypodysfibrinogenemia has features of both hypo- and dysfibrinogenemia: the reduced circulating fibrinogen levels confers a hypofibrinogenemia phenotype, and the expression of the mutation that alters functionality, a dysfibrinogenemic phenotype [6]. As with dysfibrinogenemia, in hypodysfibrinogenaemia bleeding extends from mild to moderate, but individuals are more predisposed to thrombosis.

The diagnosis of qualitative fibrinogen disorders is done by the measurement of standard clotting times, whose sensitivity depends upon the methods, reagents and coagulometers used [4]. Usually the thrombin time is prolonged, although with some variants it may be normal [5].

During fibrinogen synthesis, each newly synthetized chain is independently translocated into the endoplasmic reticulum (ER) where chaperones assist in the assembly and folding processes. Molecules are assembled in a step wise manner in the lumen of the ER: first two-chain Aα-γ and Bβ-γ complex are formed. These complexes recruit a Bβ or Aα chain respectively and form half-molecules (Aα-Bβ-γ)_1_, which in the last step dimerize through N-terminal disulphide bridges to form (Aα-Bβ-γ)_2_ hexamers [7]. Several studies performed in recombinant systems, using deletion and substitution mutants, indicate that an intact coiled-coil and correct inter- and intrachain disulphide bonds are needed for final molecular assembly [8–10]. In afibrinogenemia mutated molecules are usually absent from circulation. However, in hypofibrinogenemia or hypodysfibrinogenemia if the mutation impairs assembly variant molecules may be secreted, but underrepresented in plasma.

Here we describe a new variant with an Aα Arg104 → Cys mutation in the coiled-coil region that we have named fibrinogen Caracas IX.

## Methods

### Materials

Bovine thrombin was from Enzyme Research Laboratories (South Bend, IN). Lysine-sepharose 4B was from GE Healthcare (Piscataway, NJ). Pefabloc® SC (4- (2-Aminoethyl) benzenesulfonyl fluoride hydrochloride) was from Fluka, Sigma-Aldrich (Buchs, Switzerland). Tissue-type plasminogen activator and plasmin were from American Diagnostica (Stamford, CT). Human albumin and benzamidine were from Sigma Chemical Company (St Louis, MO). Albumin antibody conjugated to peroxidase was from Dako Corporation (Carpinteria, CA). The substrate 3,3′-diaminobenzidine (DAB) was from Thermo Scientific (Rockford, IL). The LabTek chambers and Alexa Fluor 488 were purchased from Invitrogen, Nalge Nunc International (Rochester, NY).

### Blood collection

Blood was collected in citrate (1 volume of 0.13 M trisodium citrate and 9 volumes of blood), the first 3 ml of blood discarded, and centrifuged twice at 2000 g for 10 min. The platelet poor plasma obtained (PPP) was supplemented with benzamidine 10 mM (final concentration), except the plasma to be used for fibrinolysis experiments, aliquoted and kept at −80 °C until use. Routine coagulation tests were performed with citrated plasma on coagulation analyzer STA Compact®, Stago, France. Fibrinogen level was determined by Clauss (Laboratoire Stago, Asnière, France) and clot weight method [11]. Antigenic fibrinogen concentration was measured by a latex immunoassay (Liaphen Fibrinogen, Hyphen BioMed, France).

### Mutation analysis

Blood was collected in 0.5 M ethylenediaminetetraacetate tetrasodium salt (EDTA Na_4_) (50:1). Genomic DNA was isolated using the Invisorb Spin Blood Mini Kit (Invitek GmbH, Berlin, Germany) according to the manufacturer’s protocol. Sequences comprising all exons and exon-intron boundaries from the three fibrinogen genes: FGA, FGB, and FGG were amplified by the polymerase chain reaction (PCR) according to standard protocols. After purification of the PCR products using the Invisorb Spin PCRapid Kit® (Invitek, Berlin, Germany), direct DNA cycle sequencing was performed, applying the Big Dye kit from Applied Biosystems (Foster City, CA), according to the manufacturer’s recommendations.

### Fibrinogen purification

Fibrinogen was purified essentially as described elsewhere with modifications [12]. Plasma samples were thawed and supplemented with 1 mM Pefabloc® and 5 mM EDTA (final concentrations). Plasma was depleted of plasminogen by passing through a lysine-sepharose 4B column, and then fibrinogen was purified by precipitation (×3) with 25%-saturation ammonium sulphate, pH 7.5. This fibrinogen fraction also contained co-purifying fibronectin, factor XIII and vW factor. The precipitate was dissolved in 0.3 M NaCl, dialyzed against the same solution, and stored at −80 °C until used.

The integrity of the purified fibrinogen was analyzed by sodium dodecylsulfate-polyacilamide gel electrophoresis (SDS-PAGE) on 8% gel. The coagulability of the purified fibrinogen was 96% and 93% and the yield 43 and 27%, control and patient, respectively.

### Fibrinogen degradation

Fibrinogen was incubated with plasmin as described [13] with minor modifications. Purified fibrinogen (0.9 mg/ml, in TBS) was incubated with plasmin (18 μg/ml, in TBS) in the presence of 1 mM CaCl_2_ or 5 mM EDTA at 37 °C at different incubation times (15, 30 min and 4 h), quenched with 2% SDS-DTT (v:v) sample buffer and immediately boiled. The zero time sample contained no plasmin. The fibrinogen degradation products were analyzed in a 6% gel SDS-PAGE under non reducing conditions.

### Western blotting

In order to detect fibrinogen-albumin complexes, Western blotting was performed under non reduced conditions essentially as described [14]. Briefly, purified fibrinogens (5 μg) and human albumin (5 μg) were loaded in a 5% gel SDS-PAGE, and electroblotted onto nitrocellulose [15]. The membrane was incubated for 2 h with anti-human albumin antibody conjugated to peroxidase (1:1000). The cross-reacting bands were detected with 0.6% 3, 3’diaminobenzidine (DAB), 3% cobalt chloride and 3% hydrogen peroxide.

### Activated factor XIII (FXIIIa) fibrin cross-linking

The kinetics of fibrin cross-linking was examined essentially as described elsewhere [14]. Fibrin was cross-linked by the endogenous factor XIIIa that precipitated together with fibrinogen during the purification process. Purified fibrinogen (1 mg/ml) was clotted with 1 U/ml of thrombin and 5 mM CaCl_2_. The reactions were quenched at different time points (0, 2, 5, 15 min and 1, 4, 24 h) with 2% SDS-DTT and analyzed in a 8% gel SDS-PAGE.

### Fibrin polymerization

The kinetics of fibrin formation were studied in plasma and purified fibrinogen [16]. Briefly, 100 μl fresh plasma or 0.5 mg/ml purified fibrinogen in 50 mM Tris, 0.15 M NaCl, pH 7.4 (TBS) were dispensed in a 96-well plate. Then 10 μl of 1 unit/ml bovine thrombin - 20 mM CaCl_2_ (final) was added to plasma or 5 units/ml bovine thrombin and 5 mM CaCl_2_ to fibrinogen solution. The changes in optical density (OD) were recorded every 15 s over 1 h at 350 nm in a Tecan Infinite® M 200. The polymerizations were done in three different experiments in triplicate. The lag time (s), slope (mOD/s) and final turbidity (mOD) were calculated from each curve and averaged.

### Fibrinolysis

The method was performed as described by Carter et at 2007 [17] with minor modifications. The PPP aliquots without benzamidine were used. Twenty five μl of PPP were aliquoted in a 96-well plate, then 75 μl of tPA 166 ng/ml diluted in 20 mM Hepes, 0.15 M NaCl, pH 7.4 was added. Clotting was initiated by adding 50 μl of thrombin-CaCl_2_ (0.03 U/ml and 9 mM, respectively).The OD changes were recorded at 350 nm every 15 s over 1.5 h in a TECAN® infinite 2 M microplate reader. The fibrinolysis was done at least three times in triplicate. The time to degrade 50% of the clot (T50%) was calculated from the time elapsed from half the value of the maximum absorbance of the polymerization to half-the value decreased of the maximum absorbance of the lysis curve branch. The rate of clot degradation (slope) was calculated in the descending part of the curve and the absolute value reported.

### Direct plasma mass spectrometry

Plasma was precipitated with saturated (NH_4_)_2_SO_4_ (25%, final), and the precipitate washed (2×) with 25% saturated (NH_4_)_2_SO_4_. The pellet was dissolved in 8 M urea, 30 mM dithiothreitol, 50 mM Tris–HCl, pH 8.0 and left 3 h at 37 °C. The reduced sample was injected into an Agilent 6230 Accurate-Mass electrospray time-of-flight (TOF) mass spectrometry system [18]. A Poroshell 300SB C3 (2.1 × 75 mm) column was used with an acetonitrile gradient and profile data was collected. Multi charged spectral envelopes were deconvoluted using maximum entropy processing and BioConfirm software with an isotope width of 15 Da.

### Clots biophysical characterization

In order to characterize some biophysical parameters of the clot structure, the elastic modulus, the Darcy constant (Ks), and fibrin network imaging by confocal microscopy were performed. For these experiments a healthy man with plasma fibrinogen concentration similar to the patient was chosen as a control.

### Elastic modulus

The fibrin elastic modulus (EM) was measured in the hemostasis analyzer system (HAS) Hemodyne® (Richmond, VA). Briefly, 700 μL of plasma was placed in the plastic cone and incubated for 1 min at 37 °C. Then 50 μL of a thrombin - CaCl_2_ solution (1.3 U/ml and 25 mM final concentrations; respectively) was gently and carefully mixed in. The increase in the EM was recorded every 1 min over a 30 min period. Each sample was run in triplicate in three independent experiments. The EM (kdyne/cm^2^) reported corresponds to the averaged EM value that was reached at 30 min.

### Permeation

Permeation through plasma clots was recorded essentially as described elsewhere [19]. The clotting conditions used were 1 U/ml of thrombin and 20 mM CaCl_2_ (final concentrations). The clots were left for 2 h in a moist environment at 37 °C in order to fully polymerize. The buffer percolated through the columns was TBS. Nine clots of each sample were run *per* experiment (*n* = 3) and one measurement *per* clot was taken.

The Darcy constant (Ks) was calculated using the following equation [20]:

$$ \mathrm{Ks}=\mathrm{QL}\upeta /\mathrm{tAP} $$

Where Q = volume of the buffer (in cm^3^), having a viscosity η of 0.01(poise), flowing through the column of height L (cm) and area A (cm^2^) in a given time (s) under a hydrostatic pressure P (dyne/cm^2^).

### Confocal microscopy

The experiments were done essentially as described elsewhere [16]. Briefly, clots were formed inside the eight wells LabTek chambers (Invitrogen, Nalge Nunc International, Rochester, NY). Plasma samples were mixed with Alexa Fluor 488-labeled fibrinogen (10 μg/315 μl final sample volume), then clotted with thrombin-CaCl_2_ solution (0.3 U/ml and 20 mM, respectively, final concentration). The clots were left for 2 h in a moist environment at 37 °C in order to fully polymerize.

The fibrin clots were observed in a Nikon Eclipse TE 2000 U laser scanning confocal microscopy (LSCM), with an argon ion laser (473 nm excitation and 520/540 nm for emission). The objective used was Plan APO VC 60X water immersion with a work distance of 0.27. The acquisition pinhole was set to 60 μm. Image analyses were done as described [21]. A z-stack of 60 slice was use for construct a 3D projection of 30 μm thick (0.5 μm/slice) were done. Five image by clot (212 × 212 μm) for each experiment (control and patient) were accomplished. Two diagonal lines, a horizontal and a vertical were drawn on the volumetric image of the stack using the Olympus FV10-ASW 2.1 software for obtain the pseudocolor perfil by line. Line graphs were used to calculate density (picks/μ) and diameter of fibers (μm) with Origin Pro 8 software.

### Dynamic fibrin clot growth

The spatio temporal dynamics of fibrin clot formation in real time was assessed in plasma by measuring light scattering over 30 min every 15 s using a Thrombodynamics Analyser System (HemaCore, Moscow, Russia) as previously described [21]. Briefly, plasma coagulation is activated when it is brought in contact with tissue factor coated on a plastic cuvette. The clot formation begins on the activator and propagates into the bulk of plasma in which no TF is present. Images are analyzed computationally to measure lag time, initial and stationary growth rate, size at 30 min and clot density. Based on the plots of clot size versus time, the initial velocity of clot growth is measured as the mean slope over the 2–6 min period (characterizing the VIIa-TF pathway) and the stationary velocity of clot growth is measured as the mean slope over the 15–25 min period [22].

## Results

### Case report

A new abnormal fibrinogen was discovered accidentally in an asymptomatic 11-year-old girl when routine pre-operative coagulation tests were performed for nasal turbinate surgery, which proceeded successfully. Her parents reported that when she was 3 years old she had surgery for stenosing tenosynovitis without any complications. Haemostasis work-up showed that the thrombin time was marginally prolonged, and that functional and antigenic fibrinogen levels were decreased without discrepancy in the proband and her father (Table 1). Neither parent report any hemostatic problems. Full DNA sequencing of *FGA*, *FGB* and *FGG* revealed that proband was heterozygous for a novel point mutation in *FGA* g. 3057 C > T that gives rise to an Aα Arg104 > Cys substitution (numbered without the signal peptide). In addition, she was found to be heterozygous for the Aα Ala312/Thr polymorphism. Targeted sequencing of the father showed he was also heterozygous for the novel Aα Arg104 > Cys mutation but his Aα Ala312/Thr status was not explored.

**Table 1:** Summary of coagulation tests

	Proband	Father	Reference values
TT (s)	18.6	30.8	16–18
PT (s)	15.8	54.6	13–15
aPTT (s)	34.6	42.2	27–34
Fibrinogen (Clauss)	163	122	
(mg/dl)	151		200–400
	158		
Fibrinogen antigen (mg/dl)	177	126	190–400
Factor V (%)	79	–	60–140
Factor II (%)	105	88	60–140
Factor VII (%)	79	69	65–145
Factor X (%)	82	67	65–130

*TT* thrombin time, *PT* prothrombin time, *aPTT* activated partial thromboplastin time

-: not done

### Plasma mass spectrometry

**Fig. 1:**
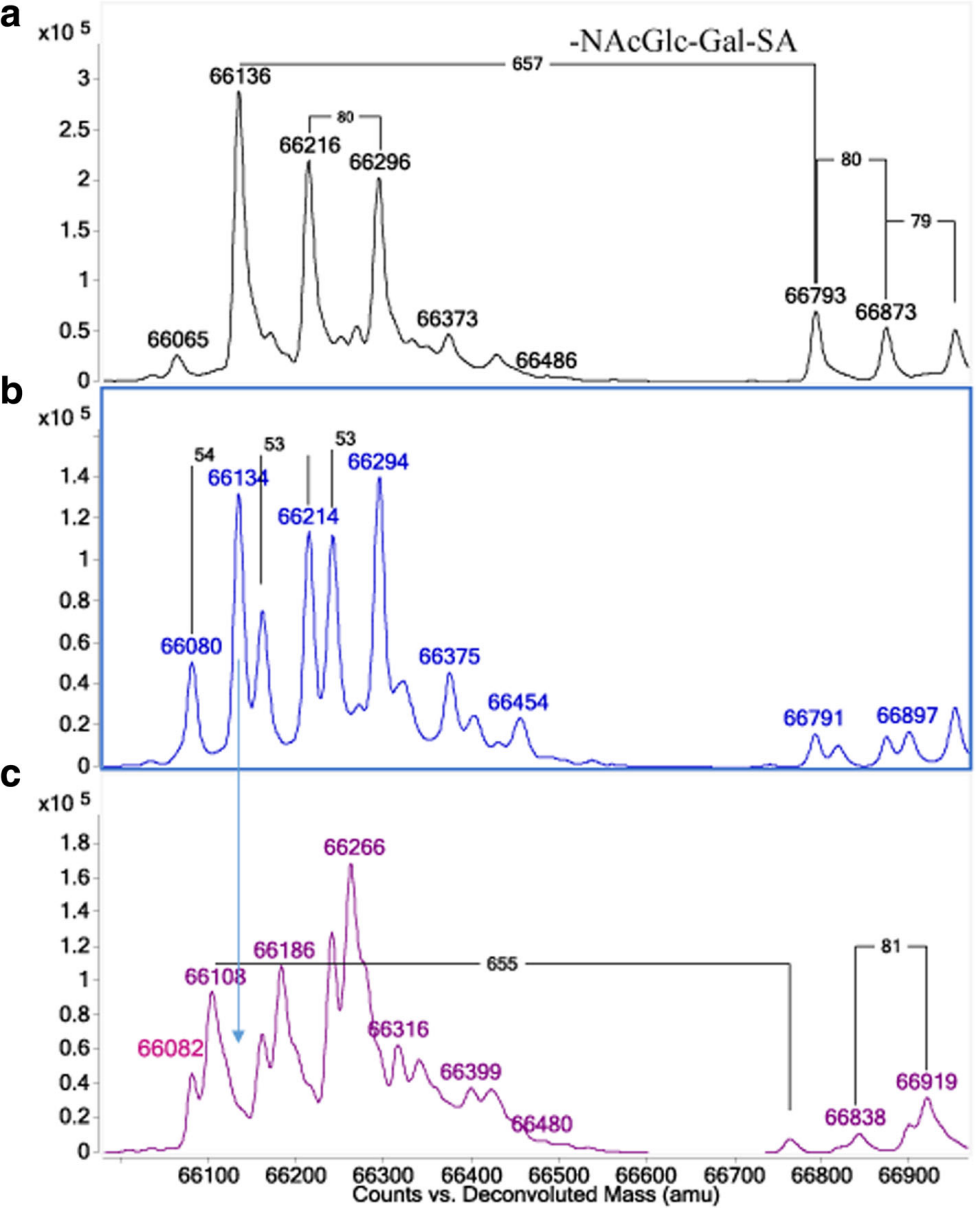
Transformed electrospray TOF spectra of fibrinogen Aα chains. **a** Normal control homozygote for the Aα Thr312 allele, (**b**) father, (**c**) proband. The father showed a normal Aα312Thr chain at 66,134 Da with a new variant chain at 66,080 Da containing an Arg → Cys mutation (−53 Da). The proband, who had no normal Aα312Thr chains, was heterozygous for the new variant and an Aα312Ala chain (66,108 Da) inherited from her mother. Peaks at +80 Da reflect successive Ser-phosphorylation. The Y- axis depicts a relative voltage response in arbitrary units

Electrospray TOF MS of purified fibrinogen from the proband and her father showed normal masses and isoforms for the Bβ and γ chain components, with no evidence of any mutations (not shown). Examination of extracted Aα chain spectra from a control, who was homozygous for the AαThr312 polymorphism, showed the expected major peak at 66,136 Da corresponding to the non-phosphorylated form of the Aα chain (theoretical mass 66,132 Da) (Fig. 1). With a peak at 66,134 Da the father appeared to have one normal copy of the AαThr312 allele and one variant copy giving rise to a protein of mass 66,080 Da. This mass decrease of 54 Da was entirely consistent with a point mutation of Arg → Cys (−53 Da). Spectra shows the proband inherited this same variant (theoretical mass 66,079 Da) from her father, together with a copy of the less common AαAla312 allele from her mother. Interestingly the variant chain with the Arg → Cys substitution was underrepresented in plasma fibrinogen and contributed only about 25% of the total Aα chain material.

Hereafter, all the studies performed to characterize the new abnormal fibrinogen were performed with the plasma of the proband’s father (indicated as patient), except the fibrin polymerisation and the fibrin clot growth assessment performed in both proband and proband’s father.

### Fibrinogen degradation, western blotting and fibrin factor XIIIa cross-linking

**Fig. 2:**
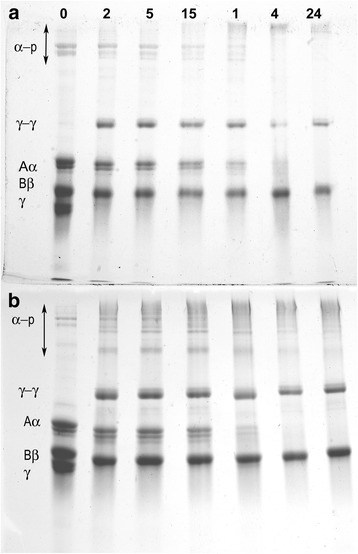
Fibrin factor XIIIa crosslinking. **a** control fibrinogen, (**b**) fibrinogen from father. Fibrin was cross-linked by the endogenous FXIII co-precipitated with fibrinogen. Polymerisation and crosslinking was initiated by addition of thrombin/CaCl_2_ and samples removed at different incubation times and run on 8%SDS/PAGE gels under reducing conditions.α-p: α-polymers

Plasmin attacks the middle of the coiled-coil of fibrinogen and generates the degradation products fragments Y and D. Surprisingly, the degradation of patient fibrinogen (Aα Arg104 > Cys) by plasmin was similar to control in the presence of either Ca^2+^ or EDTA (results not shown). In addition, the mutation introduces an unpaired –SH that could potentially form fibrinogen-albumin complexes, although by immunoblotting fibrinogen binding to albumin was negative (results not shown). The patient fibrin α-chain factor XIIIa cross-linking seemed faster compared to control. It can be seen in Fig. 2 that the patient has more intense bands of higher molecular weight corresponding to α-chain factor XIIIa cross-linking at short incubation times (i.e. 2, 5, and 15 min) compared to control.

### Fibrin polymerization and Fibrinolysis

**Fig. 3:**
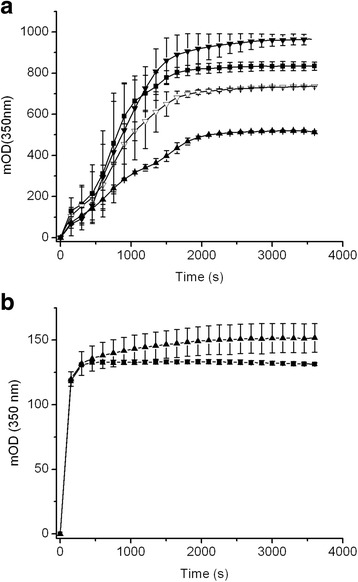
Fibrin polymerization curves. **a** fresh plasma, (**b**) purified fibrinogen. ■: control, ▼: mother, ▽: proband, ▲: father

Fibrin formation in the proband’s plasma had a near normal profile with only a slightly decreased final turbidity (~12%), while in the father the fibrin fibers growth (reflected in the slope value) and consequently the final turbidity was decreased approximately 1.3× and 40%, respectively (Table 2, Fig. 3a). With purified fibrinogen, the proband’s father polymerization was similar to control (Fig. 3b).

**Table 2:** Plasma fibrin polymerization. Plasma polymerization was done with fresh plasma. The optical density (OD) was multiplied by 1000 (mOD). The results are presented as mean (±SD)

Parameters	Control	Proband	Father	Mother
Fg* [mg/dl]	258	183	168	523
Lag time (s)	0	0	7.5 ± 11	0
Slope (mOD/s)	2.23 ± 0.19	1.66 ± 0.28	0.86 ± 0.24	2.38 ± 0.58
MaxAbs (mOD)	833 ± 18	736 ± 0.6	520 ± 4	970 ± 31

* Fg: fibrinogen, by clot weight method [11]

*MaxAbs* maximum absorbance

**Fig. 4:**
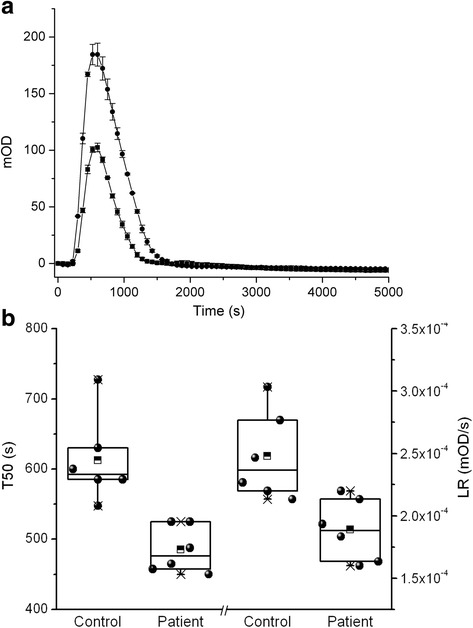
**a** Fibrinolytic process induced by t-PA. Control (•), father (■). The fathers clot was completely dissolved earlier than control. **b** Box chart of the T50 and lysis rate (LR)

The father’s fibrin clot dissolution had a slightly shorter T50: 485 ± 33 s compared to 613 ± 62 s in control (*p* = 0.001), and a slightly delayed fibrinolysis rate: 1.9 ± 0.4 × 10^−4^ OD/s compared to 2.5 ± 0.4 × 10^−4^ OD/s in control (*p* = 0.008). In Fig. 4a are shown the fibrinolysis curves, the patient area under the curve (AUC) was 1.54 × 10^7^ compared to 3.64 × 10^7^ control, approximately 2× difference, and in Fig. 4b the T50 s and slopes distribution are represented.

### Clots biophysical characterization

**Fig. 5:**
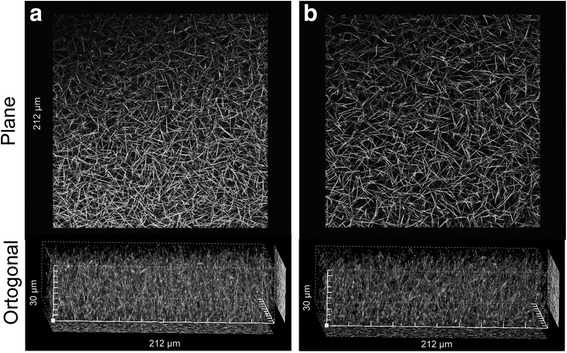
Confocal microscopy images of plasma clots. **a** Control, (**b**) Father. The fibrin was labeled with Alexa488 coupled to fibrinogen

The patient fibrin elastic modulus was approximately 500 dyne/cm^2^ less than the control, but this was not statistical significant (Table 3). The patient clot’s surface available for flux (Ks) was almost 1.8× higher than control (p < <0.001). The confocal microscopy images showed subtle differences between the patient fibrin network and control (Fig. 5). The patient fibrin density and diameter were 0.329 ± 0.016 peaks/μm and 1.150 ± 0.642 μm, respectively, compared to 0.316 peaks/μm ± 0.017 and 1.23 ± 0.02 μm in the control (*p* < 0.05). In the orthogonal fibrin clot view the patient clot looked more porous, which correlated with the porosity value (Ks) that was approximately 2× higher.

**Table 3:** Summary of clots biophysical characterization. Results are showed as the mean (±SD). In brackets the number of values averaged

	Control	Father	*p*
Fg (mg/dl)	222	176	
EM (dyne/cm^2^)	3239 ± 671 (*n* = 9)	2734 ± 534 (*n* = 7)	0.116
Ks ×10^−9^ (cm^2^)	5.1 ± 0.9 (*n* = 20)	9.4 ± 1 (*n* = 22)	1.83 × 10^−17^

Fg: fibrinogen concentration by clot weight method. EM: elastic modulus. Ks: permeation constant

### Thrombodynamics

As shown in Table 4, all the values of thrombodynamics parameters were decreased in the proband and her father compared to the healthy control. The lag time, the initial rate and the clot size at 30 min were similar between the proband and her father. The clot density was lower in father compared to the proband (9625.5 versus 11,206 arbitrary unit) reflecting his lower fibrinogen concentration. Videos of dynamic clots growth are shown in Additional file 1 (control), Additional file 2 (father) and Additional file 3 (proband).

**Table 4:** Thrombodynamics data

	Control	Proband	Father
Lag time (min)	1.1	1.45	1.4
Initial rate (μm/min)	57.45	57.5	63.3
Stationary rate (μm/min)	56.4	32.1	33.7
Size at 30 min (μm)	1814	1306.5	1368.6
Density (a.u.)	22,161	11,026	9626.5

*Min* minutes, *a.u.* arbitrary unit

## Discussion

A new fibrinogen mutation was found accidentally in an 11 year-old girl when routine pre-operative coagulation tests were performed. Gene sequencing revealed a missense mutation in *FGA*: g. 3057 C > T that predicts a p. Arg104 > Cys substitution. The low functional and antigenic fibrinogen concentrations in the father and daughter can be explained by the low expression level of the Aα Arg104 → Cys chain in their plasma fibrinogen as the new variant made up only about 25% of the total. Also, because the Aα Arg104 → Cys mutation creates a potential N-glycosylation site (Asn-Asn-Cys) centered on Asn103, mass specters were carefully examined for any new Aα chains with bi-antennary oligosaccharide side chains, but none were observed at or around their expected position at +2202 Da.

The fibrinogen coiled-coil connects the central E nodule with the distal nodule D and is made up of Aα chain residues 50–160, Bβ 81–191 and γ 24–134 [3]. At the middle of the coiled coil the structure become disorganized, probably due to the presence of proline residues at γ 70 and 76 [3]. There are 19 afibrinogenemia, 6 hypofibrinogenemia and 2 dysfibrinogenemia mutations reported in the stretch of Aα 100–112 (http://www.geht.org). At present, there are only 3 hypodysfibrinogenemia reported in the middle of the coiled coil region: Fibrinogen Epsom (Bβ Asn137_Glu141), Michigan (γ Tyr114His), and Leipzig II (Ala82Gly) [6], although the last two are compound heterozygous. These naturally occurring fibrinogen variants confirm previous findings about the role of the helical coiled coil in fibrinogen biosynthesis and secretion [10]. Other mutations in this region cause dysfibrinogenemia. For example, fibrinogen Plzen (Aα Asn106Asp) and Vizovice (Aα Phe98Ile) are characterized by low functional fibrinogen, 1.13 g/l and 1.66, but normal immunologic fibrinogen concentration 3.99 g/l and 2.89, respectively [23, 24]. The normal fibrinogen antigenic concentration excludes that these abnormal fibrinogens have impaired secretion. The low fibrinogen activity highlight the role of this part of the molecule on fibrinogen polymerization.

The kinetics of fibrin formation in plasma studied by turbidity was more impaired in the father compared to the proband. However, by thrombodynamics the father and proband behaved similarly. The results obtained using this last technique seemed more consistent with mass spectrometry. Similar polymerization kinetic would be expected for both individuals as difference of 0.15 g/l in their functional fibrinogen concentrations appeared insufficient to cause major changes polymerization. These discrepant results could result from the fact that the thrombodynamics uses a primarily different principle to initiate the coagulation cascade compared to the turbidity assay. In the former, the clotting is activated by a surface with immobilized tissue factor, which better resembles in vivo clot formation, whereas in the latter the clotting is activated by homogeneously dissolved thrombin. Fibrinogen functional correlations between these two assays require further studies.

We also expected the father would exhibit altered fibrin formation in a purified system, but probably the mutated molecules were underrepresented compared to plasma. In addition, we do not know if the mutated molecules were lost during the purification process. In the future it would be advisable for these cases to choose an immunopurification method.

The proband had higher fibrinogen concentration than her father, despite being about 5 decades younger. Different studies have found that fibrinogen increases with age [25–27]. Hager et al. reported a 25 mg/dl increases per decade [26]. Since fibrinogen is an acute phase reactant protein and CRP was not measured, it cannot be ruled out that the proband at the time of blood withdrawal could have an infectious disease.

When analyzed, an hypofibrinolysis has been observed in most hypodysfibrinogenemia [6]. The Aα Arg104 and Bβ Lys133 are the very first plasmin attack points. However, the fibrinolytic process of fibrinogen Caracas IX was close to normal. In contrast, fibrinogen Epsom with a deletion of the residues Asn137_Glu141 showed hyperfibrinolysis, together with an increased fibrinogen clearance [28]. In fibrinogen Dunedin whose γ82Ala → Gly mutation occurs near the plasmin sensitive site in the coiled coil region also displayed an increased proteolytic sensitivity. It would be interesting in the future to explore the causes of these differences in order to shed light on possibly unknown mechanisms of fibrin(o)gen degradation.

## Conclusions

The fibrinogen mutation Aα Arg104 > Cys did not introduce relevant clinical consequences probably due to its low expression levels.

## Additional files

Additional file 1: Control. (MP4 96 kb)

Additional file 2: Father. (MP4 181 kb)

Additional file 3: Proband. (MP4 113 kb)

## Abbreviations

a.u.: Arbitrary unit
aPTT: Activated partial thromboplastin time
DAB: 3, 3’diaminobenzidine
DTT: Dithiothreitrol
EDTA: Ethylenediaminetetraacetatic acid
EM: Elastic modulus
Fg: Fibrinogen
FXIIIa: Activated factor XIII
HAS: Hemostasis analyzer system
Ks: Permeation constant
MaxAbs: Maximum absorbance
OD: Optical density
PCR: Polymerase chain reaction
PPP: Platelet poor plasma
PT: Prothrombin time
SDS/PAGE: Sodium dodecyl sulfate polyacrylamide gel electrophoresis
TBS: Tris buffered saline
TF: Tissue factor
TOF-MS: Reverse phase-electrospray time of flight-mass spectrometry
tPA: Tissue type plasminogen activator
TT: Thrombin time
